# Trajectory of body mass index and height changes from childhood to adolescence: a nationwide birth cohort in Japan

**DOI:** 10.1038/s41598-021-02464-z

**Published:** 2021-11-26

**Authors:** Naomi Matsumoto, Toshihide Kubo, Kazue Nakamura, Toshiharu Mitsuhashi, Akihito Takeuchi, Hirokazu Tsukahara, Takashi Yorifuji

**Affiliations:** 1 Department of Epidemiology, Graduate School of Medicine, Dentistry and Pharmaceutical Sciences, Okayama University, 2-5-1 Shikata-cho, Kita-ku, Okayama, 700-8558 Japan; 2 Department of Pediatrics, National Hospital Organization, Okayama Medical Center, Okayama, Japan; 3 Center for Innovative Clinical Medicine, Okayama University Hospital, Okayama, Japan; 4 Department of Pediatrics, Graduate School of Medicine, Dentistry and Pharmaceutical Sciences, Okayama University, Okayama, Japan

**Keywords:** Ecological epidemiology, Obesity, Endocrinology, Risk factors

## Abstract

To investigate the dynamics of body mass index (BMI) and height changes in childhood leading to obesity in adolescents. BMI Z-scores were calculated using the LMS (lambda–mu–sigma) method based on yearly height and weight information (age 1.5–15 years) from a nationwide Japanese birth cohort that started in 2001 (n = 26,711). We delineated the trajectories of BMI and height changes leading to obesity at age 15 years using mixed effect models. Children who became obese at the age of 15 years kept relatively high BMI z-scores through childhood for both genders, and had an increasing trend over time as opposed to the normal weight group, with an increasing slope during puberty. Early adiposity rebound was associated with overweight or obesity at the age of 15 years. Age at peak height velocity (APHV) occurred earlier in the obese/overweight group at age 15 years than in the normal weight group, and occurred later in the underweight group. Obese adolescents experienced early adiposity rebound timing and maintained a serial BMI z-score increase throughout childhood, with a greater slope at puberty. An earlier peak in height gain during puberty may have contributed to the observed patterns of BMI change.

## Introduction

The prevalence of obesity has been increasing worldwide and is considered to represent a pandemic situation requiring urgent action^1–3^. In 2016, more than 1.9 billion adults aged 18 years or older (corresponding to 39% of adults) were overweight and more than 650 million (corresponding to 13% of adults) were obese^4^. The risk of all-cause mortality increases even in overweight adults: every 5 unit increase in body mass index (BMI) above 25 kg/m^2^ is associated with an approximately 31% higher risk of mortality^5^. Thus, interventions are urgently needed to reduce the prevalence of overweight and obesity. The most important intervention for obesity is prevention (especially during childhood) rather than treatment^6–8^. Simmons et al. showed that about 55% of obese children remained obese during adolescence and about 80% of obese adolescents remained obese in adulthood. Therefore, interventions to reduce and prevent obesity during childhood and adolescence are needed. Understanding BMI trajectories during development can provide useful information for prevention efforts.

The BMI trajectory during development has been evaluated in many previous studies. However, most studies focused on BMI trends in children during segmented periods such as preschool, school age, or preadolescence. Only a few large cohort studies have evaluated BMI trends longitudinally from birth to adolescence^9^. In addition, although BMI is defined as weight (in kilograms) divided by height (in meters squared), few studies have considered the role of height changes in defining BMI trajectories^10,11^. Because puberty has been reported to occur earlier in obese children, accelerated height changes during puberty should be taken into consideration to understand BMI trends during that period^12^.

Moreover, there have been considerable racial differences observed in obesity studies based on BMI^13^. Thus, BMI trajectories in various racial groups must be delineated based on large longitudinal birth cohort studies. Obese Asian individuals have been found to have higher risks of hypertension and cardiovascular disease compared with obese white Europeans as well as higher risks of early death from cardiovascular disease or any cause^14,15^. However, few studies have characterized the BMI trajectories of Asian children and their relationships with obesity during adolescence^16,17^. The Lancet World Report 2007 already highlighted the growing epidemic of obesity in Japan: obesity is a critical concern for understanding future national patterns of disease. One in four men aged 20–69 years in Japan was obese in 2000, whereas this figure had risen to one in three men by 2007^18^. Investigating the long-term BMI trends during childhood that lead to adolescent obesity is of major public health significance in Asia, especially in Japan.

The Longitudinal Survey of Newborns in the 21st Century is a national birth cohort study that has low susceptibility to cohort effects because it has been conducted for all births in Japan during specific weeks of 2001^19^. In the present study, we investigated BMI trajectories from childhood to adolescence by BMI status at adolescence using data from this large nationwide birth cohort in Japan. We examined associations between the timing of puberty (age at peak height velocity, APHV) and obesity status in adolescence.

## Methods

### Participants

The Ministry of Health, Labour, and Welfare of Japan has been conducting The Longitudinal Survey of Newborns in the 21st Century since 2001 to establish strategies to counter the declining birthrate in Japan. The survey targeted all babies born in Japan between January 10 and 17 or between July 10 and 17 of 2001. Baseline questionnaires were sent to a total of 53,575 families when eligible babies reached the age of 6 months and 47,015 families initially completed the baseline questionnaire (88% response rate). These respondents were mailed follow-up questionnaires to investigate medical conditions and behaviors when children reached the ages of 1.5, 2.5, 3.5, 4.5, 5.5, 7, 8, 9, 10, 11, 12, 13, 14, and 15 years^20–23^. Birth record data from Vital Statistics of Japan are also linked for each child participating in the study. The current study included data for children/families who responded both to the baseline questionnaire and the fifteenth questionnaire at age 15 years.

**Figure 1:**
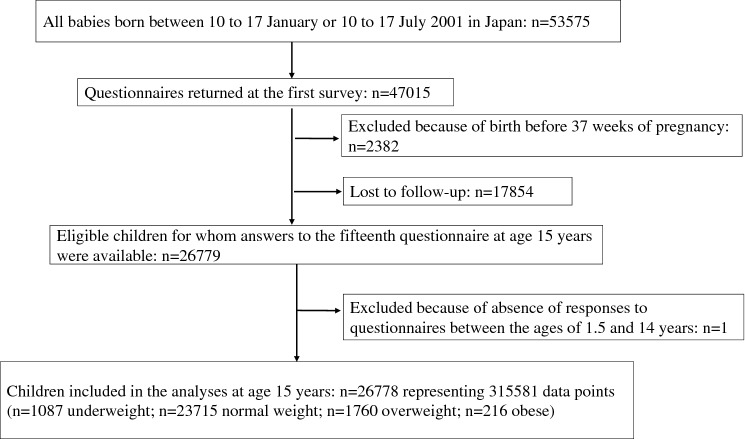
Flowchart of study participants.

The baseline survey at age 6 months included questions regarding children’s perinatal status as well as household and socioeconomic factors such as parental academic attainment, parental smoking status, and daycare attendance. The subsequent annual surveys starting at age 1.5 years included questions regarding each child’s height, weight and health status. We excluded 2382 children born before 37 weeks of pregnancy and one child with responses only for the baseline survey and the survey at age 15 years. A total of 26,778 children (315,581 data points) were included in the final analysis. A total of 11,141 children (41.61%) had responses to all 15 questionnaires between the ages of 6 months and 15 years, and responses to more than 12 questionnaires were available for the majority (91.94%) of children (Fig. 1, Table S1).

### Measures

We calculated BMI based on each participant’s reported annual height and weight. Each participant’s annual BMI was converted to a BMI Z-score using smoothed L, M, and S values for BMI standards from a representative population of Japanese children^24^. Briefly, the LMS (lambda–mu–sigma) method is a method proposed by Cole et al. to monitor changes in the skewness of the distribution during childhood as a way of constructing normalized growth standards^25^. Participants were then classified into four BMI categories based on the World Health Organization (WHO) criteria^26^: underweight (BMI standard deviation [SD] score of − 5 or more but less than − 2), normal weight (BMI SD score of − 2 or more but less than 1), overweight (BMI SD score of 1 or more but less than 2), and obese (BMI SD score of 2 or more but less than 5). The definitions of overweight and obesity were different for children under 5 years of age: a BMI Z-score of 2 SD or more was categorized as overweight and a BMI Z-score of 3 SD or more was categorized as obese. BMI category at age 15 years was the main outcome of interest in the current study.

We also calculated annual height growth for each participant by subtracting the height reported at the previous survey from that reported in the current survey. For annual height growth between 5.5 and 7 years of age, this value was multiplied by 2/3 because of the 1.5-year interval between surveys.

### Statistical analyses

We first compared baseline characteristics among the four BMI categories (underweight, normal weight, overweight and obese) at age 15 years. To evaluate potential selection bias resulting from losses to follow-up, we also compared the baseline characteristics of children included in the analysis and those of children lost to follow-up through to the fifteenth survey (at age 15 years).

We retrospectively examined annual aggregate categorical changes in individuals of the four BMI categories (groups) at age 15 years. For each group, the proportion of each BMI category at each survey between the ages of 1.5 and 14 years was calculated. In addition, we prospectively calculated the proportion of children in each BMI category at each survey between the ages of 1.5 and 14 years who eventually became underweight, normal weight, overweight, or obese at age 15 years. Note that these analyses were based on aggregate data and do not describe individual BMI changes and were performed using only the data obtained without imputation of missing values.

Under the assumption that missing data were missing at random, mixed effect models with natural cubic regression splines were applied to calculate the trajectories of BMI Z-scores and annual BMI Z-score changes through age 15 years for participants of each BMI category at age 15 years. Knots at seven locations were placed in percentiles of age to yield a sufficient number of measurements between each consecutive knot (age 1.5, 3.5, 5.5, 8.5, 11, 13 and 15 years), as recommended by Harrell^27^. The mixed effect model is useful for describing population average growth trajectories and individual growth trajectories even when data are not available for all children at all ages^28–31^. Briefly, the population average growth trajectory was modeled with fixed effects, while the individual variability is represented as random effects.

After fitting individual BMI trajectories using a mixed-effects model with natural cubic spline function, we estimated individual adiposity rebound timing as the age where the first derivative of the trajectory reached its minimum and the second derivative was positive^32^. Children were then classified into five categories (1.5–2.5 years, 3.5–4.5 years, 5.5–7 years, 8–10 years, and 11 years or older) for analysis of adiposity rebound timing^33,34^. The distribution of adiposity rebound timing was calculated for individuals of each BMI status at age 15 years overall and by gender.

Finally, we modelled annual height change and its associations with BMI status at age 15 years separately for each gender using mixed-effects models with natural cubic regression splines.

All statistical analyses were performed using Stata version 16 (StataCorp LLC, College Station, TX, USA). This study was approved by the Institutional Review Board at Okayama University Graduate School of Medicine, Dentistry, and Pharmaceutical Sciences (No.1506-073) and was conducted in accordance with the 1964 Helsinki Declaration and Ethical Guidelines for Medical and Health Research Involving Human Subjects. Informed consent was obtained by the opt-out method on the university's website.

## Results

### Demographic characteristics

Participants’ demographic characteristics according to BMI status at age 15 years are shown in Table 1. Obese adolescents tended to be boys, to be large for gestational age at birth, to live in towns or villages, to have parents with lower academic attainment, and to have mothers who smoked. During the follow-up period, 17,854 children were lost to follow-up by the fifteenth survey (at 15 years of age). Children lost to follow-up tended to have younger mothers, mothers who smoked, and mothers with lower academic attainment (Table S2).

**Table 1:** Demographic characteristics of children included in the analysis at age 1.5 years by BMI status at age 15 years (N = 26,778).

	BMI status at 15 years of age			
	Underweight	Normal weight	Overweight	Obese
	(n = 1087)	(n = 23,715)	(n = 1760)	(n = 216)
**Gender, n (%)**				
Boys	582 (53.5)	11,926 (50.3)	1022 (58.1)	133 (61.6)
Girls	505 (46.5)	11,789 (49.7)	738 (41.9)	83 (38.4)
**Birth weight, n (%)**				
< 2500 g	89 (8.2)	1278 (5.4)	81 (4.6)	7 (3.2)
2500–4000 g	994 (91.4)	22,176 (93.5)	1649 (93.7)	200 (92.6)
≥ 4000 g	3 (0.3)	258 (1.1)	29 (1.7)	9 (4.2)
**Singleton or multiple birth, n (%)**				
Singleton birth	1068 (98.3)	23,491 (99.1)	1744 (99.1)	214 (99.1)
Multiple birth	19 (1.8)	224 (0.9)	16 (0.9)	2 (0.9)
**Birth order, n (%)**				
1 (no older siblings)	556 (51.2)	11,564 (48.8)	872 (49.6)	116 (53.7)
2	390 (35.9)	8838 (37.3)	609 (34.6)	72 (33.3)
≥ 3	141 (13.0)	3313 (14.0)	279 (15.9)	28 (13.0)
**Daycare attendance at age 18 months, n (%)**				
No	957 (88.0)	19,820 (83.6)	1436 (81.6)	169 (78.2)
Yes	122 (11.2)	3660 (15.4)	296 (16.8)	45 (20.8)
**Maternal age at delivery, n (%)**				
< 25 years	81 (7.5)	2107 (8.9)	203 (11.5)	23 (10.7)
25–35 years	841 (77.4)	18,200 (76.7)	1259 (71.5)	146 (67.6)
≥ 35 years	165 (15.2)	3408 (14.4)	298 (16.9)	47 (21.8)
**Maternal smoking status, n (%)**				
No	982 (90.3)	20,841 (87.9)	1461 (83.0)	177 (81.9)
< 10/day	68 (6.3)	1921 (8.1)	180 (10.2)	16 (7.4)
≥ 10/day	30 (2.8)	843 (3.6)	105 (6.0)	23 (10.7)
**Maternal educational attainment, n (%)**				
University or higher	172 (15.8)	3850 (16.2)	202 (11.5)	35 (16.2)
Junior college	503 (46.3)	10,375 (43,8)	689 (39.2)	61 (28.2)
High school	366 (33.7)	8372 (35.3)	733 (41.7)	90 (41.7)
Junior high school or others	30 (2.8)	791 (3.3)	97 (5.5)	27 (12.5)
**Paternal educational attainment, n (%)**				
University or higher	459 (42.2)	9611 (40.5)	538 (30.6)	64 (29.6)
Junior college	172 (15.8)	3644 (15.4)	275 (15.6)	34 (15.7)
High school	372 (34.2)	8617 (36.3)	724 (41.1)	86 (39.8)
Junior high school or others	65 (6.0)	1352 (5.7)	169 (9.6)	27 (12.5)
**Residential area, n (%)**				
Wards	287 (26.4)	5080 (21.4)	340 (19.3)	42 (19.4)
Cities	625 (57.5)	14,191 (59.8)	985 (56.0)	124 (57.4)
Towns or villages	175 (16.1)	4444 (18.7)	435 (24.7)	50 (23.2)
**Infant feeding practices, n (%)**				
Formula feeding only	19 (1.8)	309 (1.3)	20 (1.1)	5 (2.3)
Partial breastfeeding	835 (76.8)	17,714 (74.7)	1327 (75.4)	182 (84.3)
Exclusive breastfeeding	228 (21.0)	5554 (23.4)	398 (22.6)	27 (12.5)

Five participants had missing birth weight information, 273 participants had missing daycare attendance information, 131 participants had missing maternal smoking information, 385 participants had missing maternal educational attainment information, and 569 participants had missing paternal educational attainment information.

### Categorical aggregate changes in each BMI status group

**Figure 2:**
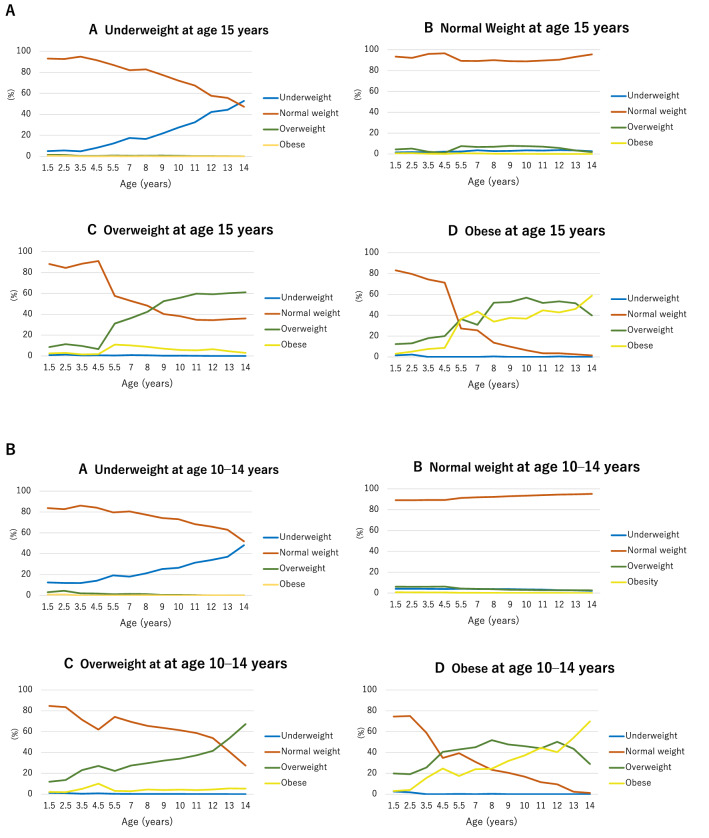
Annual categorical body mass index (BMI) changes by BMI category at age 15 years. (Panel **A**) Retrospective tracking of BMI status during childhood (age 18 months to 14 years) according to BMI status at age 15 years: underweight (**A**), normal weight (**B**), overweight (**C**) and obese (**D**). (Panel **B**). Prospective tracking of annual BMI status [underweight (**A**), normal weight (**B**), overweight (**C**), and obese **(D**)] from childhood (age 18 months to 14 years) to adolescence (age 15 years). BMI status categorization was based on the WHO definitions (under 5 years: overweight ≥ 2 SD, obese ≥ 3 SD; over 5 years: overweight ≥ 1 SD, obese ≥ 2 SD).

Figure 2A shows the results of a retrospective analysis whereby we calculated the percentages of children in the four BMI categories (underweight, normal weight, overweight, or obese) every year during childhood according to their BMI group at age 15 years. Children with normal weights at age 15 years mostly maintained normal weights throughout childhood. Although 83.1% of the children who were obese at age 15 years had normal weights at age 1.5 years, the proportion of overweight or obese children increased annually, with a large percentage of children becoming obese after age 13 years. Figure 2B shows the results of a prospective analysis whereby we calculated the proportion of children in each BMI category at each survey (from ages 1.5–14 years) who subsequently became underweight, normal weight, overweight, or obese at age 15 years. Overall, 31.0% of 7-year-old obese children had normal weights at age 15 years. The proportion of overweight/obese children who returned to normal weights by age 15 years gradually decreased, and markedly decreased after the age of 12 years. Only a small proportion of underweight/normal weight children in earlier surveys became overweight/obese at 15 years of age.

### BMI status and BMI changes during childhood

**Figure 3:**
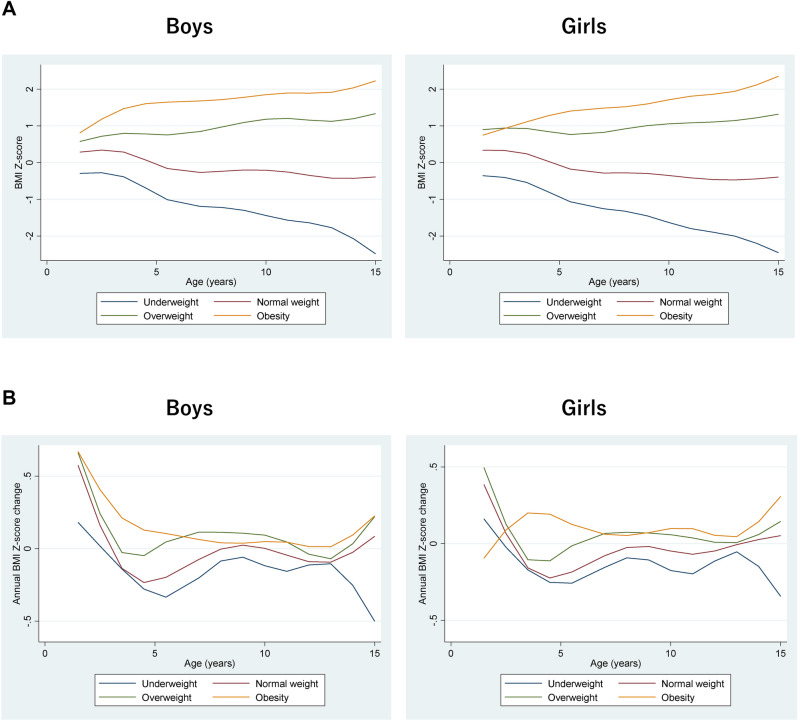
Dynamics of BMI Z-scores (**A**) and annual BMI Z-score changes (**B**) through age 15 years by gender.

The average trajectories of BMI Z-scores for boys and girls are shown in Fig. 3A. These trajectories depict the fixed effects component using mixed effects models with natural cubic splines. The average BMI Z-score trajectories of children with normal weights at age 15 years remained stable around 0 throughout childhood, whereas children who were overweight/obese at age 15 years already had relatively high BMI Z-scores by 1.5 years of age. The average trajectory for BMI Z-scores in participants who were overweight/obese at age 15 years showed a continuous increase in both genders throughout childhood, with a greater slope during puberty. Children who were underweight at age 15 years already had relatively low BMI SD scores at 1.5 years of age and, in contrast to the trajectory for participants who were obese at age 15 years, showed a marked decline in slope after puberty. Comparing the average trajectories of annual change in BMI Z-scores (Fig. 3B), participants of both genders who were obese at age 15 years showed a less pronounced dip around age 5 years than the other groups, a continuous increase in BMI Z-scores across ages, and a greater slope at puberty. By contrast, the average trajectory of annual change in BMI Z-score in participants who were overweight at age 15 years was similar to that of participants who had normal weights at age 15 years, albeit with relatively larger changes in the overweight group compared with the normal weight group.

### Impact of adiposity rebound timing

We compared adiposity rebound timing by BMI status at age 15 years overall and by gender (Table 2 and Table S3). Adiposity rebound occurred earlier in participants who were overweight/obese at age 15 years (prior to age 4.5 years) than in those who had normal weights at age 15 years. Moreover, more than 95% of participants who were obese at age 15 years, had experienced adiposity rebound before 2.5 years of age. In contrast, adiposity rebound tended to occur later in participants who were underweight at age 15 years.

**Table 2:** Timing of adiposity rebound and BMI status at age 15 years.

BMI status at age 15 years		Adiposity rebound timing							
	Total	1.5–2.5 years	(%)	3.5–4.5 years	(%)	5.5–7 years	(%)	≥ 8 years	(%)
Underweight	1087	0	(0)	0	(0)	641	(58.97)	446	(41.03)
Normal weight	23,715	362	(1.53)	4236	(17.86)	18,737	(79.01)	380	(1.60)
Overweight	1760	249	(14.15)	1509	(85.74)	2	(0.11)	0	(0)
Obese	216	208	(96.30)	8	(3.70)	0	(0)	0	(0)

### BMI status at age 15 years and APHV

**Figure 4:**
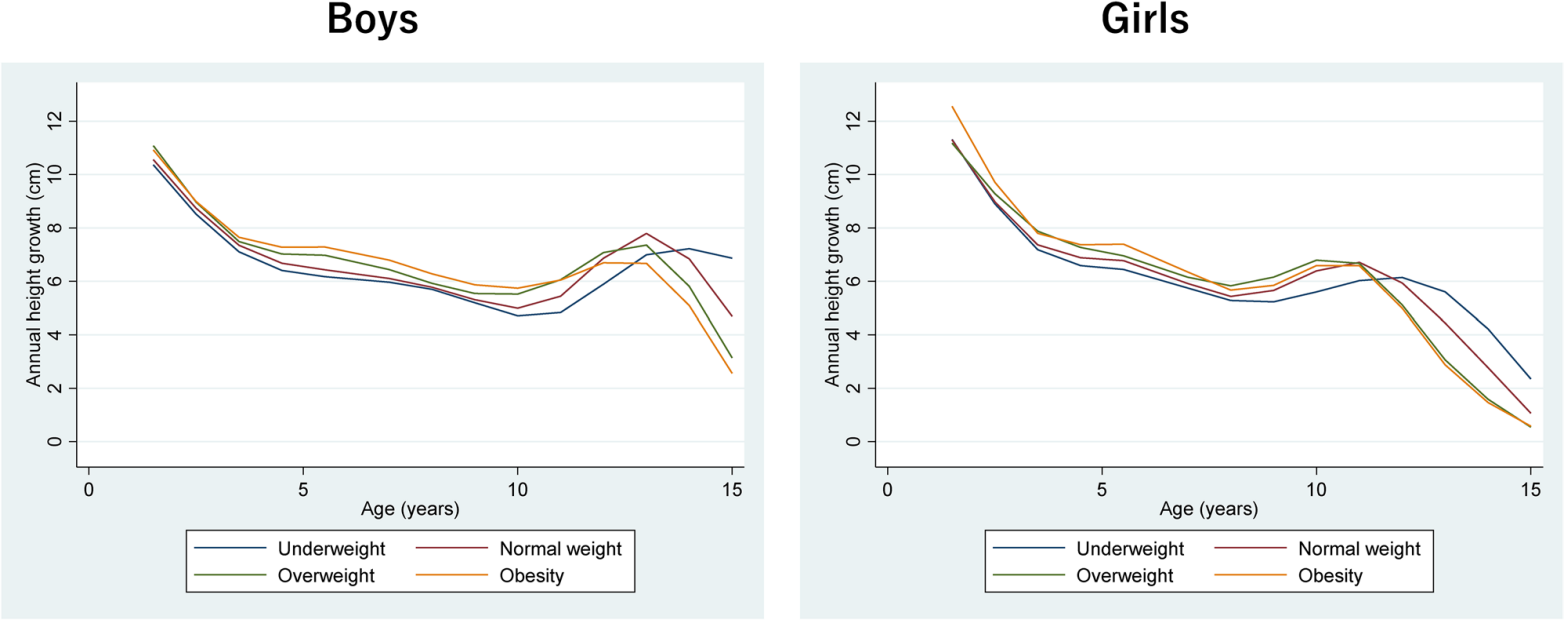
Dynamics of annual height growth (cm) through age 15 years.

Of the 26,778 participants included in the analysis, we excluded eight children whose annual height gain was never measured (i.e., no two consecutive responses). We used mixed-effects models with natural cubic regression splines to calculate the fixed-effects portion of the trajectory of annual height gain for participants of each obesity status at age 15 years, by gender (Fig. 4). Among boys, the APHV occurred earliest in participants who were obese at age 15 years, followed by those who were overweight, normal weight, and underweight at age 15 years. A similar trend was observed for girls with no marked differences between those who were obese and overweight at age 15 years.

## Discussion

In the present study, we delineated the BMI trajectories leading to obesity in adolescents and examined associations between BMI status in childhood and obesity at age 15 years using data from a large birth cohort of all Japanese children born during specific weeks of 2001. The role of annual height gain on BMI trajectories in children and adolescents was also evaluated.

Our data regarding changes in BMI during childhood are partially consistent with the findings of a German population-based study examining BMI trends from childhood to adolescence (age 15–18 years). Mandy et al. reported that BMI acceleration (i.e., a rapid increase in BMI) during childhood increased the risk of obesity in adolescence and that almost 90% of children who were obese at 3 years of age remained overweight or obese in adolescence^35^. In the present study, we found that adiposity rebound timing occurred earlier in participants of both genders who were overweight/obese at age 15 years; this difference was especially marked in those who were obese at age 15 years. The population average trajectories of BMI Z-score change among participants who were obese at age 15 years also showed an increase in BMI Z-scores over time, with no dip observed at preschool age in either gender. In our study, only 17.5% of children who were obese at age 5.5 years remained obese at the age of 15 years, and more than half of participants who were obese at age 15 years were overweight at age 13 years. Analysis of the population average trajectory for participants of each BMI status at age 15 years showed that unlike those who had normal weights at age 15 years, participants of both genders who were obese at age 15 years maintained relatively high BMI Z-scores throughout childhood, with an increasing trend over time and an increasing slope during puberty. This rapid increase in BMI Z-score during adolescence (age 14–15 years) was not observed in a previous German study. Unlike some prior studies, we included participants from a large nationwide population-based study for whom data were collected annually. In contrast with a previous German study, in which data were available for 13 or more time points in only 1% of participants, in our study the majority of children (91.74%) had responses for more than 12 surveys and 11,093 children (41.53%) had responses for all 15 surveys between the ages of 6 months and 15 years. On the basis of these comprehensive data, we were able to model BMI trajectories using multilevel models with natural cubic splines and depict the increases in BMI characteristic of obese adolescents.

Racial differences between study populations may explain some of the discrepancy observed between studies of BMI trajectories and obesity. For example, a follow-up study conducted in northern China identified a subgroup of children with a rapid increase in BMI after the onset of puberty^36^ These findings suggested that Chinese adolescents with overweight or obesity experienced BMI acceleration at two time points: at preschool ages and during puberty. Although several studies of BMI trajectories have included Asian participants, few studies have examined childhood BMI trajectories by BMI status in adolescence in a large cohort of children followed from birth until puberty. Liang et al. assessed the BMI trajectories of children aged 2–18 years using group-based trajectory modeling with random sampling from five cohorts in China. Their study mainly focused on social factors related to obesity and BMI trajectories could not be compared by BMI status in adolescence because the study included children from various ages and backgrounds. Haga et al. followed 1644 children born during an 8-year period in a district of Japan until age 12 years and identified five latent class patterns in boys and six latent class patterns in girls using latent class growth modeling. However, few large studies have longitudinally tracked BMI from birth to adolescence. The methodology used in our study would be expected to be less susceptible to cohort effects because children were born around the same time^9^.

In study of BMI, Sheila et al. assessed the influence of height gain on early adiposity rebound. BMI during puberty is expected to be affected by height gain. Several studies have shown that early adiposity rebound indicates faster growth, more advanced development, and earlier puberty^37–40^. In fact, puberty has been reported to occur earlier in obese individuals, and differences in the timing of puberty may have accentuated the increase in BMI Z-scores observed after age 13 years in obese children^10,11^. Adolescence, characterized by changes in body composition, physical fitness, and decreased insulin sensitivity during puberty, is a critical period for preventing the onset and continuation of obesity throughout the lifespan^41–43^. Ohlsson et al. showed that increased BMI through puberty and adolescence, but not in childhood, was associated with risk of adult stroke^44^. Further long-term studies are needed to assess the impact of BMI acceleration in adolescence on obesity and disease risk; at the time of BMI assessment, differences in acceleration of height growth based on childhood BMI status should be considered. Therefore, we analyzed the trajectory of annual height gain in this study. The population average trajectories for annual height gain by BMI status at age 15 years showed that APHV occurred earlier in participants who were obese/overweight at age 15 years and later in participants who were underweight at age 15 years compared with those who had normal weights at age 15 years. This phenomenon may partially explain why BMI Z-score trajectories in adolescence diverge by BMI status at age 15 years.

To date, few studies have considered the role of height when examining BMI trajectories^10–12^, especially in studies of Asian children^16,17^. Japan has been noted as a country with a rapidly growing obesity epidemic. We expect that our report will provide valuable insights for the prevention of obesity^18^.

Our study had several limitations. First, information on maternal history of obesity was unavailable. Since individual genetic predisposition and dietary habits can affect the risk of obesity^45,46^, future studies that include these data may identify additional group traits contributing to adolescent obesity. Second, we did not consider fat mass index and focused only on BMI, which may have resulted in misclassification of adiposity rebound timing^47^. However, this misclassification would likely be non-differential and bias effect estimates toward the null. Third, information on height and weight was obtained on the basis of parental reports rather than clinical measurement, which may have introduced measurement errors. Self-reported BMI may overestimate BMI in underweight individuals and underestimate BMI in overweight/obese individuals^48^. Fourth, some participants were lost to follow-up, which may have introduced selection bias. Children lost to follow-up (who tended to have younger mothers, mothers who smoked, and mothers with lower academic attainment) may have been at higher risk for overweight/obesity, and thus selection bias might have reduced the number of overweight/obese children in our study. Finally, we targeted Japanese children, which might limit generalizability to other populations.

In conclusion, our study using data from a Japanese national birth cohort showed that obese adolescents experienced early adiposity rebound timing and maintained serial BMI Z-score increases throughout childhood, with a greater slope during puberty. An earlier peak in height gain during puberty may have contributed to the observed patterns of BMI change.

## Supplementary Information

Supplementary Tables.

## Data Availability

The data that support the findings of this study are available from the Ministry of Health, Labour, and Welfare of Japan. Restrictions apply to the availability of these data, which were used under license for the current study and are not publicly available. The data used in this study are available from the authors upon reasonable request and with permission from the Ministry of Health, Labour, and Welfare of Japan.

## References

[1] Chooi YC, Ding C, Magkos F (2019). The epidemiology of obesity. Metabolism.

[2] *Prevalence and Trends Across the World Ebook.Ecog-Obesity.Eu/Chapter-Epidemiology-Prevention-across-Europe/Prevalence-Trends-across-World*.

[3] Ogden CL, Carroll MD, Lawman HG (2016). Trends in obesity prevalence among children and adolescents in the United States, 1988–1994 through 2013–2014. JAMA J. Am. Med. Assoc..

[4] Vereen RJ, Dobson NR, Olsen CH (2019). Longitudinal growth changes from birth to 8–9 years in preterm and full term births. J. Neonatal Perinatal Med..

[5] Di Angelantonio E, Bhupathiraju SN, Wormser D (2016). Body-mass index and all-cause mortality: Individual-participant-data meta-analysis of 239 prospective studies in four continents. Lancet.

[6] Pandita A, Sharma D, Pandita D, Pawar S, Tariq M, Kaul A (2016). Childhood obesity: Prevention is better than cure. Diabetes Metab. Syndr. Obes. Targets Ther..

[7] Al-Khudairy L, Loveman E, Colquitt JL (2017). Diet, physical activity and behavioural interventions for the treatment of overweight or obese adolescents aged 12 to 17 years. Cochrane Database Syst. Rev..

[8] Mead E, Brown T, Rees K (2017). Diet, physical activity and behavioural interventions for the treatment of overweight or obese children from the age of 6 to 11 years. Cochrane Database Syst. Rev..

[9] Evensen E, Wilsgaard T, Furberg A-S, Skeie G (2016). Tracking of overweight and obesity from early childhood to adolescence in a population-based cohort—The Tromsø study, fit futures. BMC Pediatr..

[10] Aksglaede L, Juul A, Olsen LW, Sørensen TIA (2009). Age at puberty and the emerging obesity epidemic. PLoS ONE.

[11] Chen LK, Wang G, Bennett WL (2021). Trajectory of body mass index from ages 2 to 7 years and age at peak height velocity in boys and girls. J. Pediatr..

[12] Li W, Liu Q, Deng X, Chen Y, Liu S, Story M (2017). Association between obesity and puberty timing: A systematic review and meta-analysis. Int. J. Environ. Res. Public Health.

[13] Deurenberg P, Deurenberg-Yap M, Guricci S (2002). Asians are different from Caucasians and from each other in their body mass index/body fat per cent relationship. Obes. Rev..

[14] Pan WH, Flegal KM, Chang HY, Yeh WT, Yeh CJ, Lee WC (2004). Body mass index and obesity-related metabolic disorders in Taiwanese and US whites and blacks: implications for definitions of overweight and obesity for Asians. Am. J. Clin. Nutr..

[15] Wen CP, Cheng TYD, Tsai SP (2009). Are Asians at greater mortality risks for being overweight than Caucasians? Redefining obesity for Asians. Public Health Nutr..

[16] Mattsson M, Maher GM, Boland F, Fitzgerald AP, Murray DM, Biesma R (2019). Group-based trajectory modelling for BMI trajectories in childhood: A systematic review. Obes. Rev..

[17] Aris IM, Chen L-W, Tint MT (2017). Body mass index trajectories in the first two years and subsequent childhood cardio-metabolic outcomes: A prospective multi-ethnic Asian cohort study. Sci. Rep..

[18] McCurry J (2007). Japan battles with obesity. Lancet.

[19] Fuse K, Nishi N, Ikeda N (2017). Cohort profile: 2001 of the longitudinal survey of newborns in the 21st century. Int. J. Epidemiol..

[20] Matsumoto N, Yorifuji T, Nakamura K, Ikeda M, Tsukahara H, Doi H (2019). Breastfeeding and risk of food allergy: A nationwide birth cohort in Japan. Allergol. Int..

[21] Yamakawa M, Yorifuji T, Inoue S, Kato T, Doi H (2013). Breastfeeding and obesity among schoolchildren. JAMA Pediatr..

[22] Kato T, Yorifuji T, Inoue S, Yamakawa M, Doi H, Kawachi I (2013). Associations of preterm births with child health and development: Japanese population-based study. J. Pediatr..

[23] Kikkawa T, Yorifuji T, Fujii Y (2018). Birth order and paediatric allergic disease: A nationwide longitudinal survey. Clin. Exp. Allergy.

[24] Kato N, Takimoto H, Sudo N (2011). The Cubic functions for spline smoothed L, S and M values for BMI reference data of Japanese children. Clin. Pediatr. Endocrinol. Case Rep. Clin. Investig. Off. J. Jpn. Soc. Pediatr. Endocrinol..

[25] Cole TJ (1990). The LMS method for constructing normalized growth standards. Eur. J. Clin. Nutr..

[26] BMI-for-age (5–19 years). Accessed September 29, 2021. https://www.who.int/toolkits/growth-reference-data-for-5to19-years/indicators/bmi-for-age.

[27] Harrell FE (2015). Regression Modeling Strategies—With Applications to Linear Models, Logistic Regression, and Survival Analysis.

[28] Andrade MAP (2015). Statistical Analysis of Human Growth and Development.

[29] Elhakeem A, Hughes RA, Tilling KM (2021). Using linear and natural cubic splines, SITAR, and latent trajectory models to characterise nonlinear longitudinal growth trajectories in cohort studies. medRxiv.

[30] Hughes RA, Tilling K, Lawlor DA (2021). Combining longitudinal data from different cohorts to examine the life-course trajectory. Am. J. Epidemiol..

[31] Gadd SC, Tennant PWG, Heppenstall AJ, Boehnke JR, Gilthorpe MS (2019). Analysing trajectories of a longitudinal exposure: A causal perspective on common methods in lifecourse research. PLoS ONE.

[32] Cissé AH, Lioret S, de Lauzon-Guillain B (2021). Association between perinatal factors, genetic susceptibility to obesity and age at adiposity rebound in children of the EDEN mother–child cohort. Int. J. Obes..

[33] Ip EH, Marshall SA, Saldana S (2017). Determinants of adiposity rebound timing in children. J. Pediatr..

[34] Baldassarre ME, Di Mauro A, Caroli M (2020). Premature birth is an independent risk factor for early adiposity rebound: Longitudinal analysis of BMI data from birth to 7 years. Nutrients.

[35] Geserick M, Vogel M, Gausche R (2018). Acceleration of BMI in early childhood and risk of sustained obesity. N. Engl. J. Med..

[36] Yuan Y, Chu C, Zheng WL (2020). Body mass index trajectories in early life is predictive of cardiometabolic risk. J. Pediatr..

[37] Kang MJ (2018). The adiposity rebound in the 21st century children: Meaning for what?. Korean J. Pediatr..

[38] Luo ZC, Cheung YB, He Q, Albertsson-Wikland K, Karlberg J (2003). Growth in early life and its relation to pubertal growth. Epidemiology.

[39] German A, Shmoish M, Hochberg Z (2015). Predicting pubertal development by infantile and childhood height, BMI, and adiposity rebound. Pediatr. Res..

[40] Marakaki C, Karapanou O, Gryparis A, Hochberg Z, Chrousos G, Papadimitriou A (2017). Early adiposity rebound and premature adrenarche. J. Pediatr..

[41] Alberga AS, Sigal RJ, Goldfield G, Prud’homme D, Kenny GP (2012). Overweight and obese teenagers: Why is adolescence a critical period?. Pediatr. Obes..

[42] Reinehr T, Roth CL (2019). Is there a causal relationship between obesity and puberty?. Lancet Child Adolesc. Heal..

[43] Schwimmer JB, Burwinkle TM, Varni JW (2003). Health-related quality of life of severely obese children and adolescents. J. Am. Med. Assoc..

[44] Ohlsson C, Bygdell M, Sondén A, Jern C, Rosengren A, Kindblom JM (2017). BMI increase through puberty and adolescence is associated with risk of adult stroke. Neurology.

[45] Lake JK, Power C, Cole TJ (1997). Child to adult body mass index in the 1958 British birth cohort: Associations with parental obesity. Arch. Dis. Child..

[46] Dotson CD, Babich J, Steinle NI (2012). Genetic predisposition and taste preference: Impact on food intake and risk of chronic disease. Curr. Nutr. Rep..

[47] Plachta-Danielzik S, Bosy-Westphal A, Kehden B (2013). Adiposity rebound is misclassified by BMI rebound. Eur. J. Clin. Nutr..

[48] Stommel M, Schoenborn CA (2009). Accuracy and usefulness of BMI measures based on self-reported weight and height: Findings from the NHANES & NHIS 2001–2006. BMC Public Health.

