# Supplementary material for: Trajectory of body mass index and height changes from childhood to adolescence: a nationwide birth cohort in Japan
Source: Sci Rep. 2021 Nov 26;11:23004. doi: 10.1038/s41598-021-02464-z (PMC8626480; doi:10.1038/s41598-021-02464-z)
Supplement: Supplementary file 1 — Supplementary Tables. [file 41598_2021_2464_MOESM1_ESM.docx]

**Article:**

**Trajectory of body mass index and height changes from childhood to adolescence: A nationwide birth cohort in Japan**

Naomi Matsumoto, MD, PhD^1^; Toshihide Kubo, MD, PhD^2^; Kazue Nakamura, MD, PhD^2^; Toshiharu Mitsuhashi, MD, PhD^3^; Akihito Takeuchi, MD, PhD^2^; Hirokazu Tsukahara, MD, PhD^4^; Takashi Yorifuji, MD, PhD^1^

^1^Department of Epidemiology, Graduate School of Medicine, Dentistry and Pharmaceutical Sciences, Okayama University, Okayama, Japan

^2^Department of Pediatrics, National Hospital Organization, Okayama Medical Center, Okayama, Japan

^3^Center for Innovative Clinical Medicine, Okayama University Hospital, Okayama, Japan.

^4^Department of Pediatrics, Okayama University, Graduate School of Medicine, Dentistry and Pharmaceutical Sciences, Okayama, Japan

| Table S1. Number of data pairs per participant | | | |
| --- | --- | --- | --- |
| Data pairs per participant | Participants | % | Cumulative % |
| 2 | 9 | 0.03% | 100% |
| 3 | 16 | 0.06% | 99.97% |
| 4 | 33 | 0.12% | 99.91% |
| 5 | 75 | 0.28% | 99.79% |
| 6 | 135 | 0.50% | 99.49% |
| 7 | 266 | 0.99% | 98.99% |
| 8 | 536 | 2.00% | 98.00% |
| 9 | 1088 | 4.06% | 96% |
| 10 | 2155 | 8.05% | 91.94% |
| 11 | 4015 | 14.99% | 83.89% |
| 12 | 7309 | 27.29% | 68.9% |
| 13 | 11141 | 41.61% | 41.61% |
| Sum | 26778 | 100% |  |
| A total of 26778 children representing 315581 data points were included in the study. One data pair consisted of birth weight information (at the first survey) and one data point during childhood (1.5 to 14 years of age) before the age of 15 years. Responses to all 15 surveys between the ages of 6 months and 15 years were obtained for 11141 children (41.61%), and responses to more than 12 surveys were obtained for the majority (91.94%) of children. | | | |

| Table S2. Baseline characteristics of eligible children included in the analysis and children lost to follow-up (N=44,633) | | | | | | |
| --- | --- | --- | --- | --- | --- | --- |
|  |  |  | Eligible children |  | Children included in the analysis at age 15 years | Children lost to follow-up at age 15 years |
|  |  |  | (n=44633) |  | (n=26779) | (n=17854) |
| Gender, n (%) | | | |  |  |  |
|  | Boys |  | 23007(51.6) |  | 13663(51.0) | 9344(52.3) |
|  | Girls |  | 21626(48.5) |  | 13116(49.0) | 8510(47.7) |
| Birth weight, n (%) | | | |  |  |  |
|  | <2500 g |  | 2468(5.5) |  | 1455(5.4) | 1013(5.7) |
|  | 2500 to 4000 g |  | 41569(93.3) |  | 25020(93.4) | 16600(93.0) |
|  | ≥4000 g |  | 520(1.2) |  | 299(1.1) | 234(1.3) |
| Singleton or multiple birth, n (%) | | | |  |  |  |
|  | Singleton birth |  | 44145(98.9) |  | 26518(99.0) | 17627(98.7) |
|  | Multiple birth |  | 488(1.1) |  | 261(1.0) | 227(1.3) |
| Parity, n (%) | | | |  |  |  |
|  | 1 (no older siblings) |  | 21952(49.2) |  | 13108(49.0) | 8844(49.5) |
|  | 2 |  | 16216(36.3) |  | 9909(37.0) | 6307(35.3) |
|  | ≥3 |  | 6465(14.5) |  | 3762(14.1) | 2703(15.1) |
| Daycare attendance at 18 months, n (%) | | | | | |  |
|  | No |  | 34866(78.1) |  | 22383(83.6) | 12483(69.9) |
|  | Yes |  | 6814(15.3) |  | 4123(15.4) | 2691(15.1) |
| Maternal age at delivery, n (%) | | | |  |  |  |
|  | <25 years |  | 5871 (13.2) |  | 2414 (9.0) | 3457 (19.4) |
|  | 25 to 35 years |  | 32899 (73.7) |  | 20447 (76.4) | 12483 (69.9) |
|  | ≥35 years |  | 5863 (13.1) |  | 3918 (14.6) | 1945 (10.9) |
| Maternal smoking status, n (%) | | | | | |  |
|  | No |  | 36665 (82.2) |  | 23462 (87.6) | 13203 (74.0) |
|  | <10/day |  | 4930 (11.1) |  | 2185 (8.2) | 2745 (15.4) |
|  | ≥10/day |  | 2731 (6.1) |  | 1001 (3.7) | 1730 (9.7) |
| Maternal educational attainment, n (%) | | | | | |  |
|  | University or higher |  | 5755 (12.9) |  | 4259 (15.9) | 1496 (8.4) |
|  | Junior college |  | 17168 (38.5) |  | 11628 (43.4) | 5540 (31.0) |
|  | High school |  | 16275 (36.5) |  | 9561 (35.7) | 6714 (37.6) |
|  | Junior high school or others |  | 2314 (5.2) |  | 946 (3.5) | 1368 (7.7) |
| Residential area, n (%) | | | |  |  |  |
|  | Wards |  | 9578 (21.5) |  | 5749 (21.5) | 3829 (21.5) |
|  | Cities |  | 26499 (59.2) |  | 15926 (59.5) | 10573 (59.2) |
|  | Towns or villages |  | 8556 (19.3) |  | 5104 (19.1) | 3452 (19.3) |
| Infant feeding practices, n (%) | | | | | |  |
|  | Formula feeding only |  | 710 (1.6) |  | 354 (1.3) | 356 (2.0) |
|  | Partial breastfeeding |  | 33991 (76.8) |  | 20058 (74.9) | 13933 (78.0) |
|  | Exclusive breastfeeding |  | 9551 (21.6) |  | 6207 (23.2) | 3344 (18.7) |
| Twelve participants had missing birth weight information, 2953 participants had missing daycare attendance information, 307 participants had missing maternal smoking information, and 3121 participants had missing maternal educational attainment information. | | | | | | |

| Table S3. Timing of adiposity rebound and BMI status at the age of 15 years by gender | | | | | | | | | | | |
| --- | --- | --- | --- | --- | --- | --- | --- | --- | --- | --- | --- |
| BMI status at age 15 years | | Adiposity rebound timing | | | | | | | | | |
|  |  | 1.5 to 2.5 years | (%) | 3.5 to 4.5 years | (%) | 5.5 to 7 years | (%) | 8 to 10 years | (%) | ≥11 years | (%) |
| Boys |  |  |  |  |  |  |  |  |  |  |  |
|  | Underweight | 29 | (4.98) | 30 | (5.15) | 227 | (39.00) | 221 | (37.97) | 75 | (12.89) |
|  | Normal weight | 1201 | (10.07) | 2839 | (23.81) | 5177 | (43.41) | 2447 | (20.52) | 262 | (2.20) |
|  | Overweight | 274 | (26.81) | 539 | (52.74) | 186 | (18.20) | 23 | (2.25) | 0 | (0) |
|  | Obesity | 72 | (54.14) | 51 | (38.35) | 10 | (7.52) | 0 | (0) | 0 | (0) |
| Girls |  |  |  |  |  |  |  |  |  |  |  |
|  | Underweight | 37 | (7.33) | 32 | (6.34) | 149 | (29.50) | 240 | (47.52) | 47 | (9.31) |
|  | Normal weight | 1688 | (14.32) | 2356 | (19.88) | 4525 | (38.38) | 2967 | (25.17) | 253 | (2.15) |
|  | Overweight | 267 | (36.18) | 275 | (37.26) | 157 | (21.27) | 36 | (4.48) | 3 | (0.41) |
|  | Obesity | 121 | (59.04) | 26 | (31.33) | 5 | (6.02) | 0 | (0) | 3 | (1.39) |
